# A Multi‐Conceptual Model Approach to Untangling the MADE Experiment

**DOI:** 10.1111/gwat.70049

**Published:** 2026-03-07

**Authors:** Joost C. Herweijer, Steven C. Young, Phil Hayes, Okke Batelaan

**Affiliations:** ^1^ ReservoirTeam P/L Mount Barker South Australia Australia; ^2^ Intera, Incorporated Austin Texas; ^3^ The University of Queensland St Lucia Queensland Australia; ^4^ College of Science & Engineering Flinders University Bedford Park South Australia Australia

## Abstract

The Macrodispersion Experiment (MADE) at Columbus Air Force Base (MS, USA) was initiated in the mid‐1980s and aimed to study solute transport in highly heterogeneous porous media by conducting large‐scale natural‐gradient tracer experiments. A review of the original field tracer experiments reveals several issues that were not addressed in most modeling efforts. These issues include: non‐stationary flow; significant questions regarding the reliability of reported hydraulic conductivity values; a significant mass imbalance (23–50%) between the injected and observed tracer; a three‐dimensional architecture based on sedimentological information; and vertical hydraulic head gradients. This paper demonstrates how these issues can be integrated into a knowledge framework that systematically assesses the knowns, unknowns, and confidence levels. Using the knowledge framework, we generate a set of multi‐conceptual models as a way forward for a holistic approach for an improved understanding of the processes that affect the interpretation of measured tracer concentrations at the MADE site. Our purpose for applying the workflow at the MADE site is twofold. First, to provide a constructive dialogue towards untangling several unresolved issues associated with modeling the MADE tracer experiments. Second, to illustrate how the application of a knowledge framework coupled with multi‐conceptual models can support a holistic approach for understanding flow and transport at highly heterogeneous sites.

## Graphical abstract

Using the MADE experiment as a case, a multi‐conceptual model roadmap is proposed for holistic assessment of solute transport including uncertainty assessment. This process involves “back to basics” review of the original MADE reports, assessment of knowns and unknowns, and the development of a comprehensive sedimentological model for aquifer architecture.

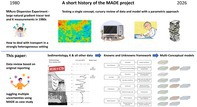

## Introduction

More than 35 years after the first MADE (macro‐dispersion) field tracer experiment (Boggs et al. 1992), the characterization of the aquifer and flow system remains a challenge for the hydrogeological community, with many aspects of tracer transport at the MADE site still poorly understood.

The MADE project at Columbus Air Force Base (Mississippi, USA) was initiated in the mid‐1980s to investigate transport in highly heterogeneous sediments (Boggs et al. 1988, 1990). The project involved the application of innovative concepts, including macro‐dispersion, geostatistics, and borehole flowmeter techniques for detailed measurement of hydraulic conductivity (K), combined with a natural‐gradient tracer test (Boggs et al. 1992). It has generated a long trail of research papers discussing various aspects of measuring and modeling tracer transport.

**Figure 1:**
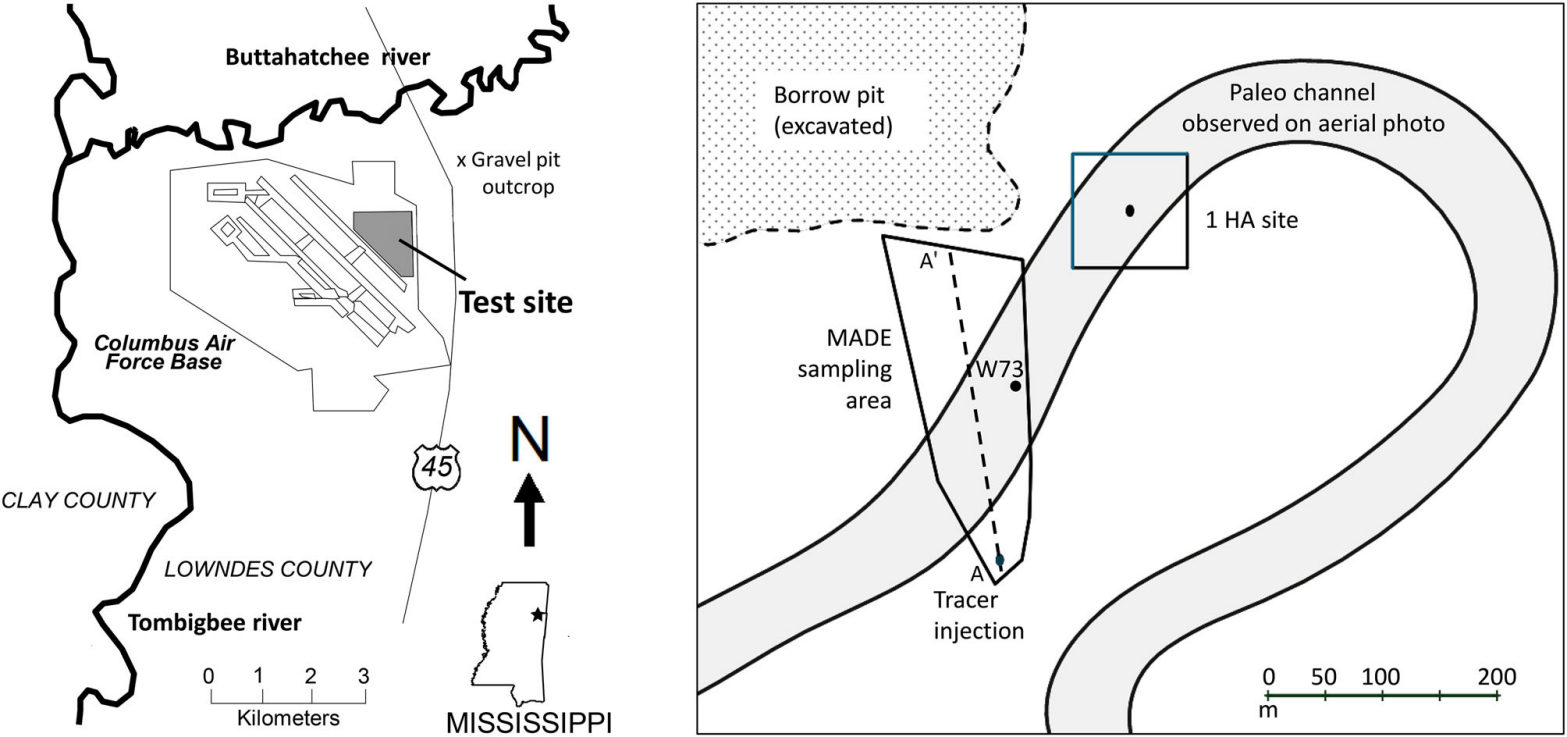
(Left) Location of Columbus Air Force Base (Mississippi, USA); (Right) Detailed view of MADE site and nearby 1 HA site.

The shallow aquifer in the Tombigbee River valley, where the MADE natural‐gradient tracer test experiments were conducted, consists of Quaternary alluvial, unconsolidated sediments overlaying bedrock of late Cretaceous age (Figure 1). An observation network spanning 350 m by 125 m was established, comprising 53 staged water level observation wells and 258 auger holes equipped with 6000 multi‐level samplers in total (Boggs et al. 1988, 1990, 1992, 1993).

Close to the MADE area, a network of 37 wells was drilled on 100 m by 100 m—the “1 HA” test site (Figure 1) (Herweijer and Young 1991; Young 1995, 1998; Herweijer 1997). At the 1 HA test site, a multitude of hydraulic tests (15 pumping with observation wells and over 100 single‐well pumping/injection/slug tests) were conducted, complemented by forced gradient experiments (two small‐scale and one site‐wide). Additional geophysical investigations, small‐scale tracer tests, and hydraulic tests were conducted at the MADE site between 2004 and 2007 (Bowling et al. 2005; Liu et al. 2010; Bohling et al. 2012).

Following the various experiments, substantial modeling has been conducted for the MADE experiment(s) from the 1990s to the present. Most of these efforts have focused on testing novel stochastic techniques to characterize heterogeneity in combination with a simplified single conceptual model, such as a stationary 2D (pseudo‐3D) flow field and the “as published” tracer distribution (e.g. Zech et al. 2021b).

The objectives of this paper are:

1. To emphasize the need for careful and rigorous “back‐to‐basics” (re‐) examination of original data.
2. To advocate for developing a 3D heterogeneity architecture based on a hierarchical multi‐scale sedimentological model.
3. To demonstrate a systematic workflow of knowns and unknowns to address the potential importance of unknowns. These unknowns include various conceptual issues which cannot simply be captured by traditional parameter uncertainty. We present this in the context of a multi‐conceptual model approach (Poeter and Anderson 2005; Enemark et al. 2019).

In this paper, we first describe the history of the MADE field experiment, including five issues of concern with respect to the MADE modeling efforts: Need to use sedimentological information, seasonal variation in water levels and hydraulic gradients, reliability of flowmeter‐based K determination, mass imbalance and representativeness of tracer observations, and modeling approach based on singular conceptualization. We then develop a knowledge framework, which is subsequently used to define a multi‐conceptual roadmap as an essential step before numerical flow modeling of a system as complex as MADE would be embarked upon.

## History of MADE Field Experiments, Interpretation, and Modeling

**Figure 2:**
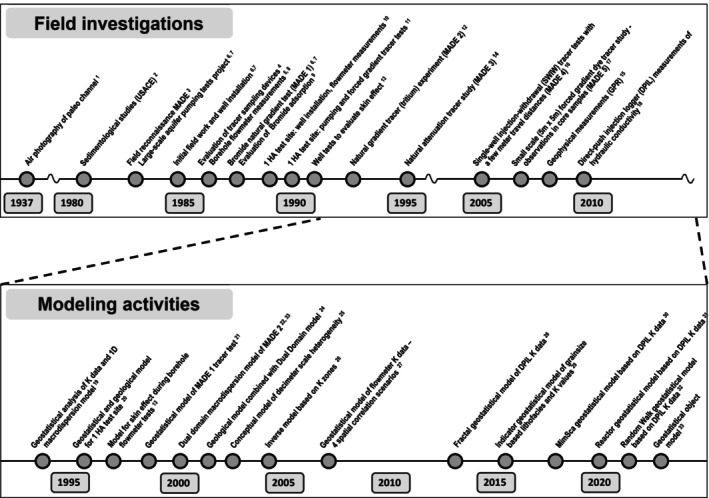
Timelines of field investigations and modeling studies relevant to the MADE site; references correspond to index numbers of timeline items.

Figure 2 shows a timeline of the field hydrogeological investigations, analysis, and modeling studies conducted at the MADE site (see Table 1 for references related to Figure 2). Included are relevant regional studies that were conducted prior to the actual MADE experiment's start in 1983. The work conducted since 1983 has been reviewed by Zheng et al. 2011 and in more recent modeling papers (Bianchi and Zheng 2016; Zech et al. 2021a, 2021b). We identify several “specific issues of concern,” that is, issues that did not receive due attention in the MADE project literature and likely have a critical impact on the interpretation of observed tracer transport at the site.

**Table 1:** References as per index in Figure 2.

Field investigations	Modeling studies
1. Farm Service Agency 1941	19. Adams and Gelhar 1992
2. Muto and Gunn 1981	20. Herweijer 1996, 1997
3. Betson et al. 1985	21. Zheng and Jiao 1998
4. Boggs et al. 1988	22. Feehley et al. 2000
5. Rehfeldt et al. 1989b	23. Harvey and Gorelick 2000
6. Boggs et al. 1990	24. Julian et al. 2001
7. Boggs et al. 1992	25. Zheng and Gorelick 2003
8. Rehfeldt et al. 1992	26. Barlebo et al. 2004
9. Boggs and Adams 1992	27. Salomon et al. 2007
10. Herweijer and Young 1990, Young et al. 1990, Young 1995	28. Dogan et al. 2014
11. Herweijer 1997, Young 1995, 1998	29. Bianchi and Zheng 2016
12. Boggs et al. 1993	30. Jankovic et al. 2017, Zech et al. 2021b
13. Young 1998	31. Fiori et al. 2019, Zech et al. 2021b
14. Stapleton et al. 2000	32. Dentz et al. 2020, Zech et al. 2021b
15. Bowling et al. 2005	33. Zech et al. 2021a, 2021b
16. Liu et al. 2010	
17. Bianchi et al. 2011	
18. Bohling et al. 2012	

### Need to Use Sedimentological Information

The first issue of concern is that sedimentological data from the MADE site, available within the literature, are seldom explicitly integrated into the conceptual or numerical frameworks to describe heterogeneity, water level observations, and solute transport. The sedimentary setting of the alluvial deposits near Columbus Air Force Base can be inferred from the regional setting (Figure 1) and is clearly visible on historical aerial imagery (Farm Service Agency 1941). It is described in studies such as Muto and Gunn (1981). This provides a basis for understanding, with limited drill data, the 3D architecture of hydraulic conductivity, which is reflected by contrasting transmissivity values from pumping tests and significant spatial variations of horizontal and vertical head gradients. However, most modeling efforts have relied on data‐intensive geostatistical representations of hydraulic conductivity heterogeneity without rigorously testing whether the resulting conductivity fields are consistent with sedimentological information and water level observations. Consequently, the link between observed hydrologic behavior and plausible sedimentological architecture remains underexplored, rather than data‐limited.

### Seasonal Variation in Water Levels and Hydraulic Gradients

The second issue of concern is the transient nature of the water levels, as well as the horizontal and vertical hydraulic water level gradients at MADE, which are evident from the piezometry data collected between December 1984 and November 1991 (Boggs et al. 1990, 1992). The modeling studies conducted for MADE typically assumed stationary flow and therefore did not account for the transient effects that potentially impacted the tracer distribution (Adams and Gelhar 1992; Zech et al. 2021b).

### Reliability of Flowmeter‐Based K Determination

Issue of concern number three entails the detailed determination of hydraulic conductivity, as this was one of the major objectives of the field campaigns (Rehfeldt et al. 1992; Liu et al. 2010; Bohling et al. 2012, 2016). The most comprehensive and “base” dataset of hydraulic conductivity values is derived from borehole flowmeter data (Rehfeldt et al. 1989b, 1992) and continues to be used as reference data (Bohling et al. 2016; Zech et al. 2021b). Several studies (Supporting Information SA) present field data and analyses that strongly suggest that the hydraulic conductivity dataset is biased and unrepresentative (Young 1998; Barlebo et al. 2004; Bianchi and Zheng 2016).

### Mass Imbalance and Representativeness of Tracer Observations

Issue of concern number four is the documented major mass imbalance of up to 50% late time deficit for the MADE 1 Bromide tracer (Adams and Gelhar 1992) and over 23% late time deficit of the MADE 2 Tritium tracer experiments (Boggs et al. 1993). Adams and Gelhar (1992) list several issues that may underlie the mass imbalance, but notably discount the possibility of “concentration smearing” between Multi‐Level Sampler (MLS) ports in individual boreholes caused by large ambient vertical head gradients as a cause for late time mass deficit.

As shown in Supporting Information SB, an initial set of small‐scale tracer tests (injecting‐withdrawing 200–400 mL tracer) indicated rapid and significant “short circuiting” of tracer among the MLS ports, but these test results were not published. Following the observed “short circuiting,” the tests were redesigned with the significantly lower tracer volume of 3.2 mL and a different injection/withdrawal protocol, resulting in a substantially more relaxed “failure criterion.” The observed “short circuiting” in the initial test provides a strong indication to suspect that MLS samplers could have introduced significant bias in the tracer concentrations measured at MADE.

Based on the assumption that the MLS had performed adequately, Adams and Gelhar (1992) extrapolated the plume concentrations beyond the coverage of the MLS network. Subsequently, they normalized the plume for the mass recovered plus the extrapolated mass. As later discussed in more detail, we consider that the various mechanisms that may have contributed to mass imbalance, including the MLS performance, warrant additional investigation to untangle the causes for significant imbalances between measured and injected tracer mass.

### Modeling Approach Based on Singular Conceptualization

Issue of concern number five is that most model studies of the MADE experiments have followed a workflow that fits with the chosen model, whether it is a traditional forward model with calibration, inversion, or geostatistical multi‐realization modeling. The chosen modeling approach has driven conceptualization, for example, a model with a single deterministic geometry (Julian et al. 2001; Barlebo et al. 2004), or a singular heterogeneity conceptualization based on a given geostatistical approach (Adams and Gelhar 1992; Bianchi and Zheng 2016; Zech et al. 2021a, 2021b). We will present a workflow that facilitates a practical approach to comprehensively assess and address uncertainty by following multiple lines of evidence, leading to a more holistic understanding of the results of the MADE experiment.

## Conceptual Model Uncertainty and Data Reliability

The timeline of field and modeling investigations reveals that various field investigations were repeated after the original investigation in the 1980s (Figure 2). One of the reasons for the follow‐up experiments was to “improve” previous measurements. However, this repeated process rather effectively captures the intrinsic inability to “know everything” and the need to recognize this “lack of knowledge,” even for a site such as MADE, where so many measurements and analyses have been conducted.

The 30 years of modeling studies at MADE demonstrate the struggle to determine “what happened.” These studies also share a common feature ‐ they treat the tracer and water level data as “best‐estimate” target data and then focus on finding a “solution” to explain these target data. Thus, these models are based (given some assumptions) on “known” concepts, parameters, and input target/calibration data (tracer and water level data). Parameter (hydraulic conductivity) uncertainty is typically addressed through sensitivity analysis (Julian et al. 2001; Barlebo et al. 2004) or realizations (Zech et al. 2021a).

### The Known, Unknown, and Uncertain

The above approach of applying numerical modeling techniques using “best‐estimate” data seems overly focused on “what is known,” whereas limited attention is paid to “what is unknown,” especially at the conceptual level. Tyson and Herweijer (2011) provide an example of how a so‐called “Rumsfeld knowledge matrix” (Marshall et al. 2019) can be applied to the modeling of oil reservoirs. One of the most important issues here is addressing “unknown unknowns,” that is, those issues we do not know that we do not know them. These can be pursued through rigorous and lateral thinking, combined with creatively challenging existing knowledge, while avoiding the pitfalls of anchoring and groupthink. Another important category is the “unknown knowns” or “things we don't know that we know,” that is, “forgotten” and “overlooked issues.” These are also described as “the silent presuppositions that we are not aware of” (Žižek 2006). It is important to recognize the possibility of “unknown knowns” and make the utmost effort to limit this category by expanding the “known knowns” via the rigorous study of pre‐existing work, including knowledge emanating from unrelated studies or other disciplines.

To characterize the state of knowledge about the MADE aquifer system and the major tracer experiments, MADE 1 and MADE 2, we adopt the following knowledge classification:

- Known: implies that something is unambiguously supported by data, measurements or other information, given a well‐defined error margin.
- Plausible: data, measurements, or information strongly indicate a given parameterization (within a well‐defined distribution or options) or concept.
- Unknown/Uncertain: there is a best estimate parameterization or conceptualization with a substantial range of error and alternatives that must be considered.
- Unreliable: there is a clear indication that the data are flawed and unrepresentative.

**Figure 3:**
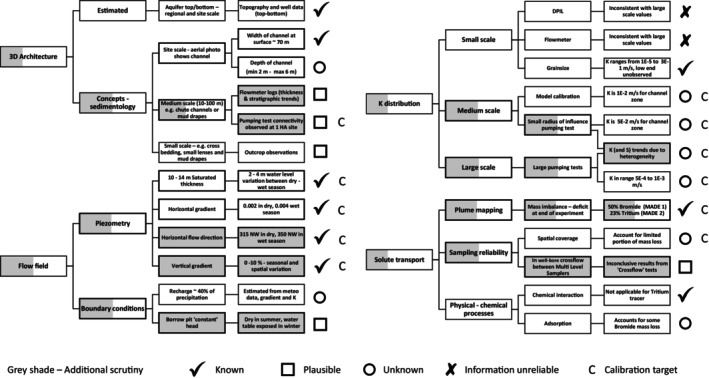
Knowledge framework for the MADE experiment, showing for each of the elements of the major knowledge components Geology and aquifer geometry, Flow field, K distribution, and Solute transport what is known, plausible, unknown/uncertain, or unreliable. The gray shading indicates whether knowledge elements merit additional scrutiny via modeling. [Correction added after first online publication on 16 March 2026: Figure 3 has been updated.]

Figure 3 presents an analysis and classification of the state of knowledge. This knowledge framework follows a typical workflow for aquifer assessment, splitting up major components (aquifer geometry, flow field, hydraulic conductivity, and tracer transport) into subcomponents that align with the MADE project. Gray highlighting indicates knowledge that we believe merits additional scrutiny. For each type of knowledge, Figure 3 indicates how it fits with the above‐presented classification.

### 3D Heterogeneous Hydraulic Conductivity Architecture

With grainsize and clay content as the primary controls of hydraulic conductivity, sedimentology is a key candidate for establishing a 3D architecture for a heterogeneous aquifer (Weber 1982; Rogiers et al. 2013). The most common approach is the assessment of lithofacies, which leads to the identification of sedimentary structures and depositional processes, ultimately resulting in a sedimentological model (Miall 1988). The sedimentary architecture encompasses a range of scales, providing a hierarchical framework for modeling, as applied to oil and gas reservoirs (Weber and van Geuns 1990) and aquifers (de Marsily et al. 2005).

For the MADE site, we consider the following scales: regional (>1000 m), site (100–1000 m), meso (10–100 m), and small (<10 m). Whilst the choice of these scales is arbitrary and somewhat flexible, this choice is fit for practical purposes, and hence, it is included in the knowledge framework (Figure 3).

At the regional and site scales, the geometry of the MADE shallow aquifer is defined by the top of the aquifer (topographical surface) and the bedrock Eutaw formation, which is classified as an aquitard. Between the upstream injection area and the downstream section of the test site, the aquifer thickness increases by about 25% (Boggs et al. 1990). From a regional perspective, it is also clear that the modern alluvium consists of deposits by the meandering Tombigbee River (Figure 1).

**Figure 4:**
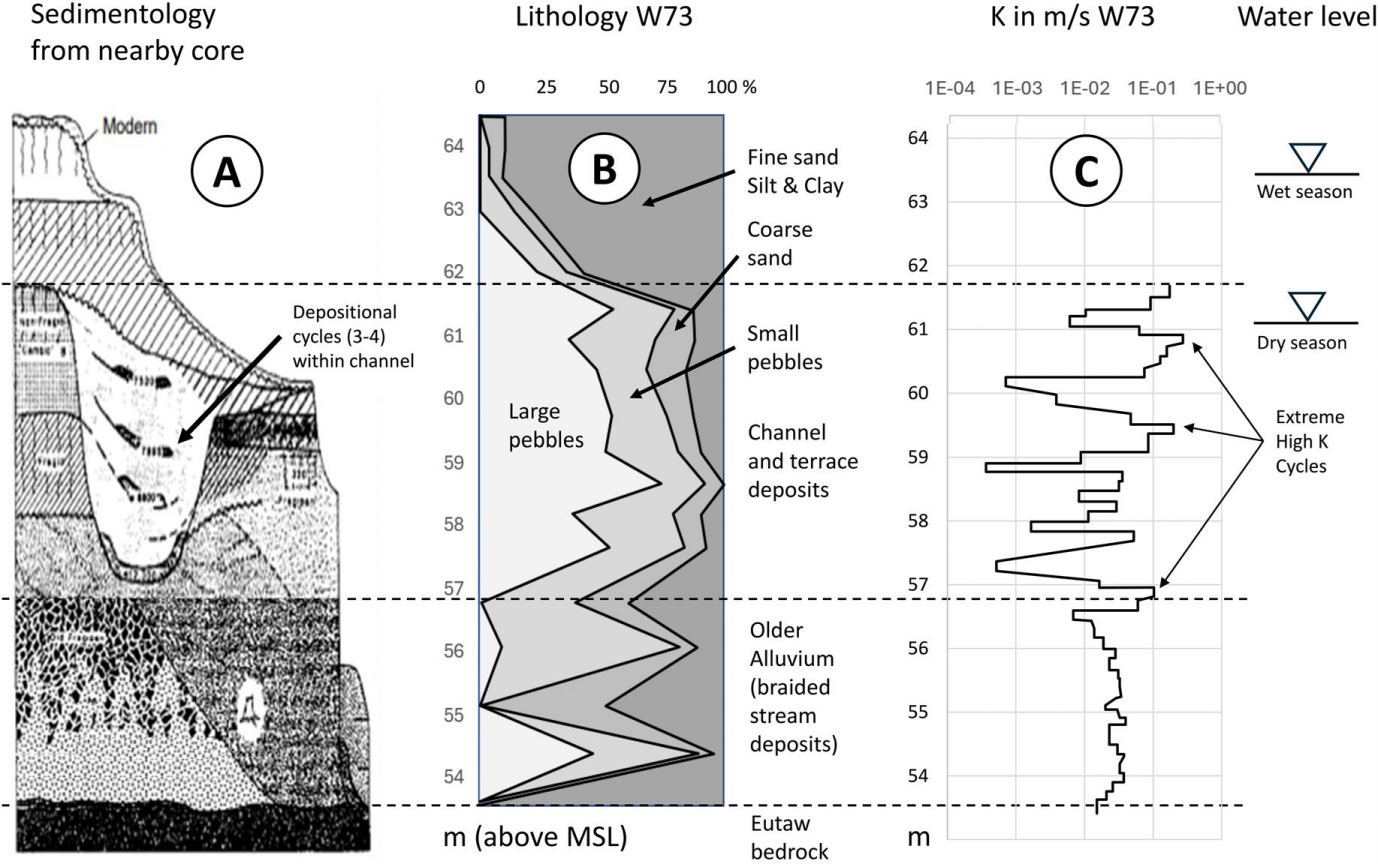
(A) Core/outcrop section after Muto and Gunn (1981), (B) Soil log of well W73, (C) Borehole flowmeter log of W73 (see Figure 1 for location). Note the depositional cycles in the sedimentological core description (A), which are reflected by K variations in the borehole flow meter log (C).

At the “site scale,” a meander of a paleo‐channel is observed through air photography (Farm Services Agency 1941). In a 1956 aerial photograph, the outline of the former river meander (Figure 1) is particularly evident due to differences in color and growth of newly developed vegetation. A comprehensive archaeological study of the Tombigbee Valley was conducted on behalf of the US Army Corps of Engineers (Muto and Gunn 1981) and presented detailed geological and sedimentological studies of the Quaternary floodplain deposits, including two core holes and a sedimentological transect located within 20 km south of the MADE site (Figure 4a). Figure 4b is a soil log of MADE well W73 located in the channel and indicates that the top part of this channel (surface at 62 m MSL) is an oxbow clay, overlaying a pebble‐dominated channel deposit down to 57 m MSL, overlaying mixed fine‐coarse older alluvium. The sedimentological profile and soil composition closely correlate with the borehole flowmeter‐derived hydraulic conductivities as obtained for the well W73 (Figure 4c), which are not part of the MADE databases because it was conducted as part of the research project associated with the 1‐HA test site (Young 1998).

**Figure 5:**
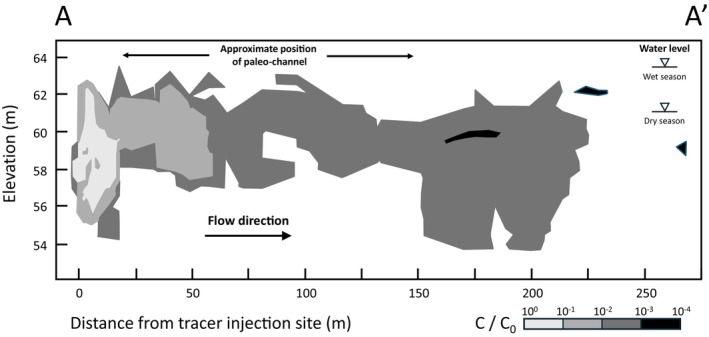
Tritium concentration (relative to initial concentration *C*_0_) along cross‐section A–A′ (see Figure 1 for location and Figure 6 for sedimentological context), showing at 20 m distance from the injection site upward movement into the high K zone associated with the channel (after Boggs et al. 1993).

The width of the channel on the aerial photograph is 70 m. Using a width‐thickness relation for analogue highly sinuous, meandering rivers (Leeder 1973), a 4–8 m thickness is estimated for the channel. This thickness estimate from the analogue data is consistent with the channel bottom at 57 m MSL, halfway to the alluvial aquifer depth (Figure 4). This range challenges the realism of the 2 m channel thickness inferred by Bowling et al. (2005) from geophysical GPR measurements, the assumption that the channel fully extends to the bottom of the aquifer, as made by Julian et al. (2001). The channel (as an elevated hydraulic conductivity zone) is further confirmed by pumping tests (Boggs et al. 1992; Young 1995; Herweijer 1997), borehole flowmeter measurements (Rehfeldt et al. 1989b, 1992; Young 1995), the change of hydraulic gradient (Boggs et al. 1990, 1992), and a vertical cross‐section of the Tritium plume (Figure 5).

**Figure 6:**
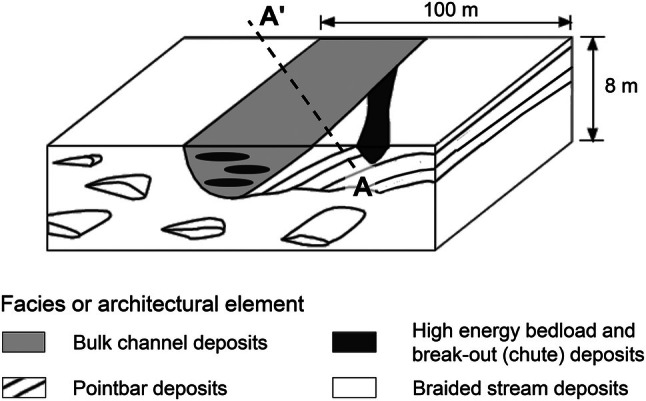
Conceptual sedimentological model for alluvial aquifer at Columbus Air Force Base (after Herweijer 1996). The dashed line indicates the approximate position of section A–A′ along the length of the MADE site (Figures 1 and 5) relative to the block diagram.

Major and unexplored geological aspects for MADE are meso‐scale (10–100 m) sedimentary features. Figure 6 illustrates the depositional framework (meandering channel), including site and meso‐scale features as developed by Herweijer and Young (1991). This depositional framework combines stratigraphic and hydraulic conductivity information with a sedimentary analog presented by Muto and Gunn (1981) and McGowen and Garner (1970). This block diagram was derived from the 1 HA test site data and is directly applicable to the MADE site (Figure 1).

The meso‐scale features encompass:

- Sedimentary cycles (Figure 4A) within the channel, consisting of coarse gravel bedload material (high‐energy deposits) topped by finer material (low‐energy deposits), concur with hydraulic conductivity variations in the borehole flowmeter log (Figure 4C).
- Chute channels deposited during high flows, resulting in elongated very high hydraulic conductivities at the inner edge of the meandering channel and possibly cutting into the point bar (Figure 6).
- Braid bars in the lower part of the alluvial aquifers (Figure 6). These are typically on the smaller end of the meso‐scale and, due to the less stable depositional environment, are more variable in grainsize.

The importance of these meso‐scale features in creating high hydraulic conductivity pathways is confirmed via pumping and tracer tests at the 1 HA site (Herweijer and Young 1991; Young 1995).

At the small‐scale end of the spectrum are lenticular features with a length of 2–10 m and a thickness of 0.1–1 m identified in soil mapping of a quarry face near the MADE site (Rehfeldt et al. 1989a, 1992).

### Groundwater Flow Field and Hydraulic Gradients

**Figure 7:**
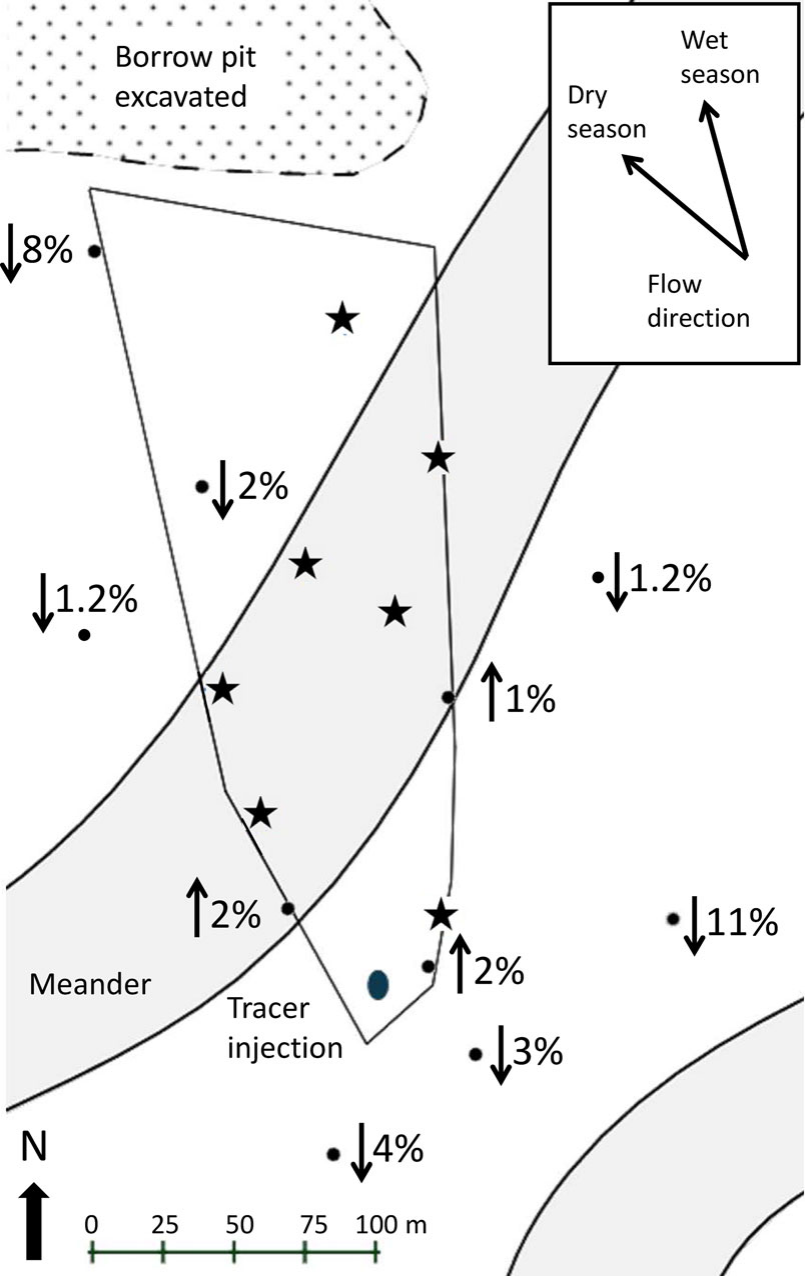
Average upward ↑ and downward ↓ vertical head gradients as a percentage of the vertical piezometer separation observed in the period 1986–1989 (stars denote gradient <0.5%).

The comprehensive piezometric data set allows observations regarding the flow field that has been “known” since the early days of the MADE site investigations (Figure 3). However, the importance of this “known” data set has been much underutilized in subsequent studies. Boggs et al. (1990, 1992) describe the following characteristics of the flow field:

- The occurrence of significant ambient vertical head gradients (Figure 7) indicates vertical flow mostly at the edges of the paleo‐channel (as reflected by the tracer distribution in Figure 5) in combination with low vertical hydraulic conductivity, for example, due to silt/clay intervals (Figure 4a and 4c) within and on top of relatively high hydraulic conductivity deposits.
- The orientation of the horizontal hydraulic gradient changes by 35°, between the wet winter (December–March) and dry (summer) seasons. As a result, the flow direction is approximately in the direction of 300° WNW during 8 months of the year, which is a deviation of 50° from the general direction of the tracer observation network, which is 350° N (Figure 7).
- Significant seasonal variation is observed in the elevation of the water table, which is 3–4 m higher in the wet winter than in the dry summer, increasing the aquifer saturated thickness by over 30% (Figure 4).

The vertical head gradients shown in Figure 7 were obtained from piezometer bundles installed in the observation boreholes. Such a bundle typically consists of a piezometer completed in the upper portion of the aquifer and another piezometer completed in the lower portion. The vertical head gradient is based on a 5 m difference between the lower and upper observations.

The general trend of the vertical head gradients (Figure 7) is downward between the southern arm of the channel and the injection site, upward from the injection site to the edge of the northern channel arm (which runs across the MADE network), and downward north of the channel. This trend is consistent with the flux of water being evenly distributed across the whole aquifer, being forced upward through the channel part of the aquifer, which has a much higher hydraulic conductivity, and subsequently forced downward north of the channel. This effect is also evidenced in the vertical section along the flow direction through the tracer plume (Figure 5).

The influence of the borrow pit (Boggs et al. 1990), directly at the north end of the MADE site (Figure 1), is unknown and may act as a boundary condition. It is assumed that this pit was excavated to provide building material for the Columbus Air Force Base. There is no information on the assessment of the hydrologic impact of this borrow pit. It is quite possible that water seeps into this pit during the wet season's high water table conditions, which would explain the northward rotation of the flow field towards the borrow pit during the wet season. During the dry season, the water levels are probably below the bottom of the borrow pit, and the flow direction is more to the southwest in the direction of the Tombigbee and Buttahatchee Rivers (Figure 1). Both the WNW flow direction and the large drop in water level during the 8‐month‐long dry season could affect the plume observations, as the tracer could flow outside the observation network and the tracer could be “stuck” in the unsaturated zone, both via perching and/or as residual capillary retention.

### Hydraulic Conductivities

**Figure 8:**
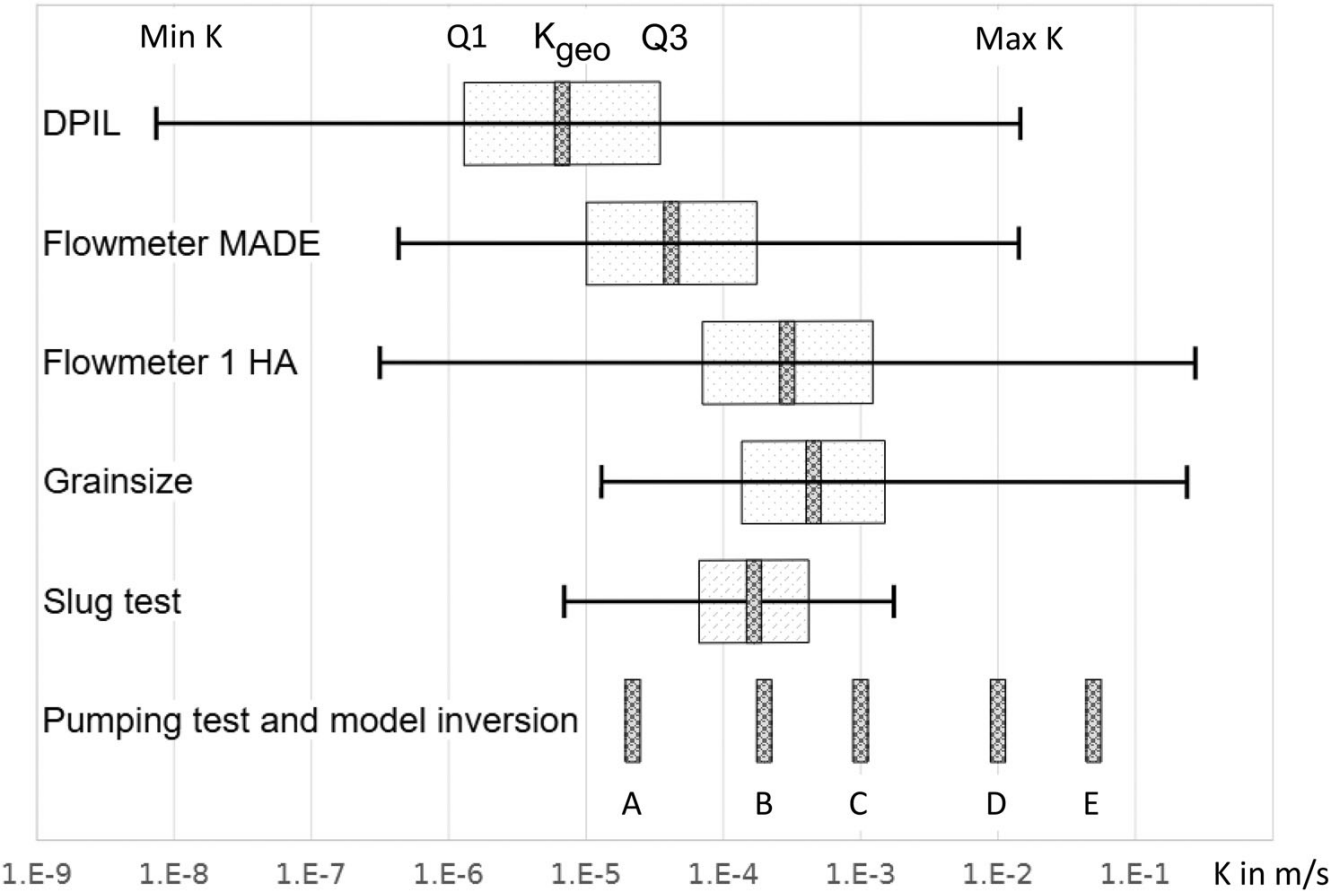
Horizontal hydraulic conductivities at MADE and 1 HA site (after Zech et al. 2021a, with some additions). DPIL (Direct Push Injection Logger—Bohling et al. 2016); Flowmeter MADE (Rehfeldt et al. 1992); Flowmeter 1 HA (Young 1998); Grainsize, Slug test, Pumping test A and B (Boggs et al. 1990); C—Site wide pumping test 1 HA site (Young 1995); D—Model inversion for channel zone (Barlebo et al. 2004) and pumping test in channel zone 1 HA site (Herweijer and Young 1990); E—Site wide pumping test 1 HA site, early time derivative analysis (Herweijer 1997).

Figure 8 presents the range of hydraulic conductivity measurements at MADE and the nearby 1 HA site based on six types of tests or datasets. It shows that the hydraulic conductivity is strongly heterogeneous as it varies over several orders of magnitude. The six hydraulic conductivity datasets represent different volumes of aquifer because of distinct testing procedures in combination with the actual aquifer properties.

Despite the extensive use of borehole flowmeter and DPIL (Direct Push Injection Logger) data for modeling (Zech et al. 2021b), misconceptions persist regarding the validity and representativeness of these data. The DPIL method is a highly localized injection test in which the pressure is observed 0.4 m away from the injection point (Bohling et al. 2012). Assuming some anisotropy, the volume of aquifer material “tested” using DPIL measurements would extend 0.3–1 m from the injection well. Grainsize‐derived hydraulic conductivities are also highly localized, as the material analyzed comes from the wellbore. This means a volume with a lateral dimension of about 0.1 m (wellbore diameter) and, depending on the drilling/sampling technique/interval, a vertical dimension of 0.3–1 m. Borehole flowmeter‐derived hydraulic conductivities are laterally affected by the radius of influence of the pumping tests used to determine the transmissivities from which the hydraulic conductivities are calculated (Young 1995; Bianchi 2017). Practically, the radius of influence is in the order of several to 10+ m.

The effective hydraulic conductivities obtained at MADE range from 3E‐5 to 1E‐2 m/s. This variability likely reflects the location of the test and the type of analysis. The lowest value was obtained from a test conducted at the southern end of the MADE site, outside the channel area. The highest values (0.01–0.1 m/s) are derived from pumping tests that focus on early‐time drawdown and represent the channel area (Herweijer 1997), and from an inverse numerical model (Barlebo et al. 2004) that includes distinct zones coinciding with the channel deposit.

As previously observed by Barlebo et al. (2004) and evident from Figure 8, neither the MADE borehole flowmeter nor the DPIL measurements appear realistic vs. the large‐scale effective K values from pumping tests and model calibration. The geometric average and upper and lower quartile values of the distributions are well below most of the large‐scale effective K values. In addition, there are two (D and E) large‐scale effective K values at and beyond the largest values of the MADE borehole flowmeter and DPIL K distributions.

As further discussed in Supporting Information SA, Young (1998) investigated the apparent lack of high‐end values in the MADE flowmeter K measurements and concluded that a low K skin effect is a major issue for wells in the paleo‐channel, which were typically backfilled with clay‐dominant material from the top layer. Rehfeldt et al. (1989) calculated K by applying the Cooper‐Jacob equation using two data points, the initial water level and the water level during stationary pumping. As shown by Young (1991, 1998), analysis of a full transient drawdown curve obtained with transducers allows for isolating the skin effect and determining K from the late time Cooper–Jacob slope. The latter represents the aquifer K, unaffected by low‐K skin, and yields systematically higher K values in the very coarse sediments of the paleo‐channel deposits. This method was also applied to the flowmeter K data at the 1 HA test site and several check wells at MADE (Young 1998).

Figure 8 also shows that the K distribution from grainsize analysis and flowmeter measurements at the 1 HA better represents the effective large‐scale K values from a point of view of geometric averages, quartiles, and maximum value. Hence, from a distribution point of view, these measurements are more appropriate as model input. The latter was also assumed by Bianchi and Zheng (2016), who used hydraulic conductivities derived from grainsize analysis as a basis for assigning K values to lithofacies. It is a concern that recent papers still rely on the original flowmeter hydraulic conductivity data set for MADE (Rehfeldt et al. 1992) as a “benchmark” for hydraulic conductivity estimation (DPIL method, Bohling et al. 2016) and model input (Zech et al. 2021b).

### Tracer Concentrations

Both the MADE 1 Bromide tracer test and the MADE 2 Tritium tracer test exhibit in early time an estimated tracer mass up to 200%, and in late time a mass deficit of more than 50% of the MADE 1 Bromide tracer (Adams and Gelhar 1992) and over 23% of the MADE 2 Tritium tracer experiments (Boggs et al. 1993). Adams and Gelhar (1992) attribute the initial mass surplus to in‐wellbore cross flow due to preferential flow through high K zones combined with the injection vertical head gradient, resulting in high concentration “fingers” of the plume smearing across the wellbore and hence being over‐sampled. Boggs and Adams (1992) present several studies suggesting that 17% of the injected Bromide mass may have been lost from the groundwater system because the low pH promoted bromide adsorption. This leaves over 33% of the Bromide mass deficit unaccounted for. Adams and Gelhar (1992) assume that there is no “vertical concentration smearing bias” in boreholes with MLS ports. Hence, they apply extrapolation and mass renormalization to correct for partial mass recovery during late time. Most modeling papers (Bianchi and Zheng 2016; Zech et al. 2021b) use this “normalized” plume obtained by Adams and Gelhar (1992).

As elaborated in Supporting Information SB, we consider that there is a distinct possibility that the previously discussed natural vertical head gradients (Figure 7) could cause ambient vertical flow in the annulus of MLS‐equipped boreholes and hence significantly bias tracer sampling.

Given the probability of biased sampling of tracer concentrations, the effect of water level change in the saturated thickness by 30%, and the rotation of the flow field away from the network, the plume configuration obtained via extrapolation and normalization by Adams and Gelhar (1992) is not necessarily a realistic representation of the tracer plume. As discussed later, the multi‐conceptual model approach is employed to investigate the following three hypotheses regarding the mass imbalance of the injected tracer, thereby contributing to a holistic understanding of the MADE experiments.

1. During the later phase of the tracer experiment, low concentration fingers of the freshwater recharge would move through a preferential path of a high hydraulic conductivity lens, and subsequently, the vertical head gradients would “smear” that low concentration across the bore, and hence, low concentrations would be oversampled, resulting in a deficit of observed mass.
2. The tracer observations are affected by the significant change in water table of nearly 3 m between the wet winter and dry summer seasons. For the channel, this top section of the aquifer consists of a considerable fraction of fines and clays, resulting in moisture retention and the possibility of localized perched water when the water table is significantly deeper during the dry season.
3. The significant rotation of the flow field during the dry season in a direction 35° towards W away from the N–S axis of the MADE site may also lead to tracer “escaping the observation network.”

### Use of the Tracer Data for Model Testing

Most papers have attempted to “match” the above‐mentioned and normalized plume using a variety of (mostly stochastic) heterogeneity models (Bianchi and Zheng 2016; Fiori et al. 2019; Zech et al. 2021a, 2021b; de Lange 2025). This approach utilizes only part of the information, specifically the normalized plume, which in the case of the Bromide test accounts for only half of the mass injected, and for the Tritium test, 77% of the mass injected. As discussed in the following sections, the above approach overlooks other observations and measurements that are highly indicative of heterogeneity, such as vertical head gradients and pumping tests with a non‐homogeneous response (Herweijer and Young 1991; Herweijer 1996).

## Multi‐Conceptual Model Roadmap

To untangle the complexity of the MADE site and the hydrogeological observations, we propose a forensic process where all lines of evidence are followed without being anchored on “a priori” assumptions. This process also needs to include a keen awareness of the limited understanding (unknowns) concerning conceptual issues and the interpretation of various measurements.

In this section, we will lay out a multi‐conceptual model roadmap that could be investigated via modeling efforts directed to capture a broad spectrum of options. Since the 1980s, this approach has been extensively and successfully applied to geology, fluid flow, and recovery from subsurface oil and gas reservoirs (van Elk et al. 2000; Smith et al. 2004; Bentley and Smith 2008). For groundwater, examples of this approach are outlined by Refsgaard et al. (2012) and Enemark et al. (2019). Note that the word “conceptual” is meant here in its broadest sense and includes geology, model structure (layering and gridding), boundary conditions, assumptions regarding sampling bias, development of aquifer properties, choice of input parameters (validity, alternatives), and so on.

Table 2 presents a set of multi‐conceptual models applicable to the MADE experiment. As the main subdivision of this table (first column left), we use the “knowledge” categorization as in the knowledge framework (Figure 3).

1. “Known” issues must feature in any model, certainly if the objective of the model is to simulate the field experiment.
2. “Plausible” issues feature a “best‐estimate” interpretation that still needs to be checked vs. an alternative interpretation that would also fit the data.
3. “Unknown/Uncertain” issues should be addressed by the creation of models designed to investigate the potential importance of key but unvalidated assumptions.

**Table 2:** Multi‐Conceptual Model Roadmap Versus Selected Models for MADE. Gray Shading Indicates High Priority.

No.	Info type	Multi‐model issues …gray shade—high priority	Adams and Gelhar (1992)	Zheng and Jiao (1998)	Harvey and Gorelick (2000)	Feehley et al. (2000)	Julian et al. (2001)	Zheng and Gorelick (2003)	Barlebo et al. (2004)	Salamon et al. (2007)	Dogan et al. (2014)	Bianchi and Zheng (2016)	Fiori et al. (2019)	Zech et al. (2021a, 2021b)
**1**		**Knowns—must be considered**												
1.1	A	Geometry of aquifer including observed top and bottom topography of the aquifer and existence of borrow pit directly North of the test site		Mostly implemented		Mostly implemented	Mostly implemented		Mostly implemented		Mostly implemented	Mostly implemented		
1.2	A	The meander feature observed at the surface and related flatter hydraulic gradient					Included		Partial‐high K zone					Partial‐K contrast
1.3	B	Large seasonal variation of saturated thickness, vertical gradients and flow direction—MADE 1 and MADE 2 experiments started in different seasons												
**2**		**Plausible multi‐model options—should be tested vs alternative hypothesis (opposite)**												
2.1	A/C	The depth and hydaulic conductivity of the meander deposit (plausible: mid aquifer depth and strongly elevated hydraulic conductivity)		Partially as per flowmeter K data		Partially as per flowmeter K data	High K across full depth		High K zone to mid aquifer					High K full depth
2.2	C	The most reliable small‐scale K measurements (A: Grainsize values; B: Borehole flowmeter values per Rehfeldt et al. 1992; C: DPIL per Bohling et al. 2016; D: other)	Flowmeter (A)	Flowmeter (A)	Flowmeter (A) + adjustment	Flowmeter (A)	Zone K values via calibration (D)	Effective K for 3E‐4 gradient (D)	Zone K values via inversion (D)	Flowmeter (A)	Flowmeter (A) and DPIL (C)	Grainsize (A)	Flowmeter (A) and DPIL (C)	Flowmeter (A) and DPIL (C)
2.3	D	Correction of the tracer test data for mass balance error (A: As‐is field data; B: Total mass adjustment (normalizing per time snapshot) and averaging into 1D plume per Adams and Gelhar 1992; C: Other averaging/adjustment)	A: As‐is MADE 1 field data and 1D adjusted (B)	As‐is MADE 2 field data (A)	As‐is MADE 1 and 2 field data (A)	As‐is MADE 2 field data (A)	Maximum along vertical (C)	As‐is MADE 2 field data (A)	As‐is MADE 2 field data (A)	1D and 2D mass adjusted MADE 2 data (A, C)	1D Adjusted MADE 1 plume	1D adjusted plume (B)	1D adjusted plume (B)	1D adjusted plume (B)
2.4	D	In wellbore cross‐flow occurs between multi‐level samplers given observed vertical gradients (plausible) vs. multi‐level samplers provide correct tracer data												
**3**		**Uncertainty—define base and end member cases**												
3.1	B	The impact of the borrow pit on flow direction during the wet season where it potentially exposes the water table and functions as a “constant head.”												
3.2	A	The size and configuration of site‐ and meso‐scale high hydraulic conductivity streaks	Correlation range (partial)	Correlation range (partial)		Correlation range (partial)				Correlation range (partial)	Correlation range (partial)	Correlation range lithofacies	Correlation range (partial)	Correlation range (partial)
3.3	A	The presence of thin finite meso‐scale clay/mud layers												Low K inclusions
3.4	D	Tracer adsorption and dual domain exchange vs. absence of these processes			Single and Dual domain	Single and Dual domain	Single and Dual domain	Molecular diffusion						
3.5	B	Retention of tracer in unsaturated zone during dry season (either capillary or perching on low hydraulic conductivity lenses—see Nimmo et al. 2017)												
3.6	B	Effect of seasonal flow direction change on ability of network to capture tracer plume							Network capture investigated					
3.7	A	Size and nature small‐scale (<10 m lateral) geological/hydraulic features.	Macrodispersion	Correlation range stochastic realizations	Macrodispersion	Correlation range stochastic realizations		0.1 m scale with 2 K‐ratio options		Correlation range stochastic realizations	Correlation range stochastic realizations	Correlation range stochastic realizations	Correlation range stochastic realizations	Correlation range stochastic realizations
3.8	B	Effective recharge as a portion of precipitation		Steady state		Steady state								
3.9	B	Nature of flowfield in relation to recharge and K	2 options		2 options									
		Multi model issues investigated	5	6	5	6	6	4	6	4	5	5	3	7
		Priority multi model issues investigated	2	2	1	2	3	1	4	2	3	3	0	3
	A, B, C, and D—see text for explanation		Disclaimer: the comparison of these modeling efforts is judgemental and only intended to be indicative											
			“Correlation range” and “Stochastic realizations” generalizes that different methods take into account a measure of spatial correlation and employ Monte Carlo analysis											

In the second column, we indicate the “information type” along the lines of the main subdivision of the knowledge framework (Figure 3).

- Aquifer geometry: large, meso, and small‐scale architecture
- Flow field (spatial and temporal), including boundary conditions
- Hydraulic conductivity and its relation to scale
- Tracer transport and mapping, reliability, and physico‐chemical processes

Similar to the normal procedure for establishing a single conceptual model, some professional/engineering judgment is needed to implement the above methodology, especially when it comes to different interpretations of non‐numerical information, such as sedimentology and boundary conditions, in combination with hydrological non‐uniqueness.

### Practical Implications of Multi‐Conceptual Model Analysis

Table 2 shows 16 issues that underlie the multi‐conceptual model process. The first three issues (1.1–1.3) are “knowns” and hence must be considered in a model analysis. For the next four issues (2.1–2.4), we have identified plausible options and potential alternatives that could be considered. The remaining nine issues (3.1–3.9) are “unknown” and could be analyzed via base and end‐member cases to assess the impact of the “unknown.”

The above‐outlined multi‐conceptual model approach could result in a substantial modeling workload. To keep the workload manageable, we propose a prioritization scheme (gray‐shading in Table 2) and the application of relatively simple models or analytical calculations focused on specific issues. For example, a model representing a small area around a single borehole could be used to design and interpret small‐scale tracer tests using only 3.2 mL of injected tracer to evaluate the potential for crossflow between adjacent MLS ports. This model could then be used to simulate the tests presented by Boggs et al. (1988) and impose the observed vertical hydraulic gradients to analyze their effects on causing unnatural vertical migration of groundwater within the backfilled material between MLS sampling ports. Similarly, a small representative section of MADE could be modeled to assess the effects of seasonal water level changes and the potential retention or loss of tracer in the unsaturated zone or perched above low vertical hydraulic conductivity lenses.

### Multi‐Conceptual Model Roadmap Compared to Previous Modeling Approaches

The right‐hand side of Table 2 shows an overview of key modeling efforts conducted for MADE. Of the 16 multi‐conceptual model issues, only 10 have been looked at in previous modeling efforts, and of the nine priority issues, only four have been investigated. Two papers consider 6 of the 16 multi‐conceptual model issues and four of the nine priority multi‐conceptual model issues (Julian et al. 2001; Barlebo et al. 2004).

None of the modeling efforts have simulated the transient nature of groundwater flow at MADE, that is, the large seasonal variation in unsaturated thickness and the significant seasonal change in flow direction. Another key issue, the presence of the paleo‐channel combined with the flatter hydraulic gradient, has been incorporated into only a few models. Many models also omit including the substantial thickening of the MADE aquifer downstream from the injection area.

Some of the unknowns and multi‐conceptual models in Figure 3 and Table 2 are partially addressed by earlier‐developed models. For example, the model developed by Barlebo et al. (2004) demonstrated the discrepancy between the effective channel hydraulic conductivity and the borehole flowmeter‐derived hydraulic conductivity (2.2 in Table 2) and concluded that the higher grainsize‐based hydraulic conductivities provide a more representative distribution of small‐scale K‐values than the borehole flowmeter (and DPIL—Direct Push Injection Logger) K values. Barlebo et al. (2004) also tested the hypothesis whether the tracer observation network causes plume truncation (3.6 in Table 2) at late time. The dual‐domain model (Feehley et al. 2000) does provide a pointer that there are elements in the hydrogeological architecture in which tracer transport is much faster than in other elements, assuming the tracer test observations are “correct.” This model points to 3.5 in Table 2, where the tracer could be “held” up in the unsaturated zone.

A considerable number of papers consider the small‐scale geological/hydraulic features (3.6 in Table 2), typically using geostatistical or stochastic techniques that enable an ensemble assessment of the effect of spatial variability on the tracer flow. As indicated in Table 2, this small‐scale spatial variability is a secondary issue vs. the larger (meso) heterogeneity. All papers recognize the mass imbalance in the MADE 1 and MADE 2 tracer tests (2.3 in Table 2). Most authors make a single choice in “fixing” this problem via the previously discussed spatial averaging and temporal renormalization of tracer observations (Adams and Gelhar 1992) without testing any alternative scenario that could have a significant impact on the mass balance (2.4, 3.5, and 3.6 in Table 2).

Another issue is that none of the models have been specifically tested against the observed vertical head gradients, which are some of the most suitable data for calibration, especially with respect to heterogeneity. For example, the models proposed by Bianchi and Zheng (2016) and Zech et al. (2021a, 2021b) contain significant hydraulic conductivity contrasts within a vertical profile. The validity of these models could be easily tested by assessing the modeled vertical head gradients, and whether these are within an order of magnitude of those observed at MADE.

## Discussion

Burt and McDonnell (2015) state in their paper, “Whither field hydrology? The need for discovery science and outrageous hydrological hypotheses,” that modeling is a very powerful tool. However, it must be applied in direct synthesis, with appreciation for field data and conceptual exploration. In our survey of the MADE literature, we found such “discovery” often lacking, along with a general lack of scrutiny of information related to data collection and analysis, including site and regional geology, aquifer hydraulic properties, and concentration data.

One of the major absentees in the published history of the MADE project is an assessment of the aquifer architecture via sedimentological analysis. Rehfeldt et al. (1989a) state that the “aquifer at the MADE site … is clearly a braided‐stream deposit (pp. 2–23)”. After publication of the aerial photo showing a meander (Herweijer and Young 1991), Rehfeldt et al. (1992) and Bowling et al. (2005) argued that the paleo‐channel outlined in Figure 1 should not be used to guide the development of a site conceptual model, as they argued that channel deposits are surficial and have a limited impact on groundwater flow. However, this is contradicted by the lower horizontal hydraulic gradient observed in the part of the MADE site where the paleo‐channel is observed and the vertical head gradients at the edges of the channel.

Regarding the use of sedimentological data we note the following:

- Existing regional geological studies offer numerous potential clues that can be combined with published analogues (sedimentary and geomorphological) to characterize the features and scales of heterogeneity. For example, a simple review of Google Earth reveals major geomorphological features, such as the abandoned meanders from paleo‐channels in the Tombigbee Valley near the MADE site. Drillings and borehole flowmeter logs confirm the relevance of such a feature at the MADE site (Figure 4). Following the knowledge framework outlined in this paper, this information can then be collated with site‐specific data from grainsize logs, pumping tests, and so on.
- Publications are available on quantitative hierarchical sedimentological models for characterizing heterogeneous oil and gas‐bearing formations (Weber and van Geuns 1990; Herweijer et al. 2000; Ringrose and Bentley 2015), presenting techniques and concepts applicable to characterizing heterogeneity in aquifers. Using such techniques, groundwater models based on a hierarchical sedimentological architecture are, for example, presented by Fogg et al. 1998; de Marsily et al. (2005), Ronayne et al. (2008), Alloisio et al. (2011), and Dowling et al. (2013). The commonality of these oil and gas and groundwater applications is that sedimentology is used to characterize the heterogeneity of an aquifer/reservoir based on a limited data set. For MADE, the hydrofacies approach, as presented by Bianchi and Zheng (2016), is the closest equivalent. However, it only models a single level of heterogeneity and relies heavily on a large number of drill holes to establish the hydrofacies and K distribution.
- The heterogeneity that impacts the MADE plume emerges at multiple spatial scales. As well established for petroleum reservoirs (Flint and Bryant 1993; Fryberger et al. 2011) and groundwater aquifers (Fogg and Zhang 2016), this multiple‐scale heterogeneity is the rule rather than the exception and often unique to a geological setting. Hence, “standard representative levels of heterogeneity at a single scale” (Zech et al. 2023) may not be an appropriate concept to reliably underpin solute transport description. Rather, there is a need for site‐specific geologically based heterogeneity assessment and modeling as described above.

The low‐biased Rehfeldt et al. (1989b, 1992) and DPIL (Direct Push Injection Logger ‐ Bohling et al. 2016) hydraulic conductivity values were incorporated into many modeling studies (e.g. Zech et al. 2021a, 2021b). This raises again the question whether careful review of field collection methods and previous literature is an undervalued undertaking. Several papers present field data and model findings (Young 1998; Barlebo et al. 2004; Bianchi and Zheng 2016), raising serious doubts about the representativeness of the Rehfeldt et al. (1992) hydraulic conductivity data. These doubts are most prevalent in the bedload deposits of the paleo‐channel, which are quite relevant to transport. The DPIL measurements (Bohling et al. 2012) were benchmarked against these unrepresentative original flowmeter measurements (Rehfeldt et al. 1992) and hence are also questionable, if not invalid.

Another key issue in this context is that, while testing the reliability of MLS‐equipped boreholes (Boggs et al. 1988), multiple small‐scale tracer test methods and “pass” criteria were considered. After the first series of tests exhibited significant spreading along the MLS, the test procedure was adjusted by significantly reducing the tracer volume. Reviewing the original reporting on this matter, we consider it questionable whether Boggs et al. (1988) sufficiently demonstrated, using a 3.2 mL tracer slug, that cross‐communication between sampling ports that are spaced 15 cm apart in a single borehole under the influence of vertical hydraulic head gradients, which often were greater than 5%, is within acceptable limits to support the presumption that the MSL samplers worked adequately.

The above, in combination with other issues such as the transient flow field, are factors relevant to the large mass imbalance between the released and observed amount of tracer. Remediating this imbalance via “extrapolation, averaging and normalisation” (Adams and Gelhar 1992) is questionable. Hence, especially for the MADE 1 (Bromide) tracer test, any “match” between models and these averaged and normalized tracer data (Zech et al. 2021b) is suspicious and could be just a “spurious correlation”.

## Conclusions and Recommendations

First and foremost, 35 years of investigations have shown that modeling highly heterogeneous sites, such as the MADE site, requires a holistic hydrogeological approach that examines various concepts from multiple angles and cannot be “solved” by looking at one issue in isolation or using a single “magic bullet” approach or algorithm. Perhaps eagerness to readily apply recently developed modeling techniques and novel algorithms has contributed to a multitude of modeling approaches being applied to the MADE experiment without first performing a thorough vetting of the field data collection methods and sufficiently integrating the field data in order to check, and if appropriate, challenge the conceptualization of the site's geology and hydrology.

We also note that statements regarding the collection and analysis of field data as published in the initial papers (Adams and Gelhar 1992; Boggs and Adams 1992; Boggs et al. 1992; Rehfeldt et al. 1992) have served as a benchmark for MADE and have been reused by many modeling studies without performing independent reviews of the original project publications and related studies performed at or near the MADE site. We strongly recommend that researchers carefully scrutinize original field reports and cast a wide net to find additional data, such as public domain reports on geology.

As shown in this paper, a 3D heterogeneity architecture comprising nested scales forms a template for understanding the spatial distribution of the hydraulic conductivity, which can be easily diagnosed using a sedimentological model and water level data obtained from limited data (drill holes). This is in contrast to the geostatistical (macro‐dispersion) approach that requires a lot of data or is constrained by mostly unsupported spatial continuity assumptions reflecting a single scale.

We have proposed refreshing the narrative by posing different questions based on a “known/unknown/uncertain” framework, leading to a set of multiple conceptual models (including scenarios on data validity). Based on this, we developed a roadmap for advancing a holistic understanding of the key hydrogeologic issues, including a multi‐scale sedimentological model and a detailed analysis of water level data to assess tracer transport at the MADE site. It demonstrates a systematic workflow to address the potential importance of unknowns, resulting in a need to use multiple conceptual models and to critically evaluate field testing methods. A firm understanding of unknowns is necessary and often undervalued in modeling. Hence, we recommend a thorough assessment of the unknowns to feed a modeling approach based on multi‐conceptual models.

## Supporting information

A: Hydraulic conductivity values determined from borehole flowmeter data ‐ Validity of data as reported by Rehfeldt et al. (1989 and Rehfeldt et al. 1992).
B: Representativeness of the tracer concentrations from the multi‐level samplers used at MADE.

## Data Availability

The data that support the findings of this study are available from the corresponding author upon reasonable request.
