# Supplementary material for: A Multi‐Conceptual Model Approach to Untangling the MADE Experiment
Source: Ground Water. 2026 Mar 7;64(2):130–47. doi: 10.1111/gwat.70049 (PMC12990960; doi:10.1111/gwat.70049)
Supplement: Supplementary file 1 — A: Hydraulic conductivity values determined from borehole flowmeter data ‐ Validity of data as reported by Rehfeldt et al. (1989 and Rehfeldt et al. 1992). B: Representativeness of the tracer concentrations from the multi‐level samplers used at MADE. [file GWAT-64-130-s001.pdf]

Supplementary information A

Hydraulic Conductivity Values Determined from Borehole Flowmeter Data – Validity of data as reported by Rehfeldt et al. (1989 and 1992)

The hydraulic conductivity values developed by Rehfeldt et al. (1989 and 1992) have been relied on for many studies involving the MADE site, both in modelling studies up to a few years ago (Zech et al. 2021) and as ‘truth benchmark’ for alternative hydraulic conductivity determination (DPIL method, see Bohling et al., 2016). However, Young (1998) and Young et al. (1998) identify significant sources of bias/error in the hydraulic conductivity values for the borehole flowmeter as calculated by Rehfeldt et al. (1989 and 1992).

Young (1998) and Young et al. (1998) conducted flowmeter tests at seven pairs of closely-spaced wells located at the MADE and 1HA site (Figure 1 main paper). Each pair included an augered well installed using a ‘natural backfill’ per the method used by Rehfeldt et al. (1989 and 1992) and a well installed using a gravel pack material. For the wells inside the paleo-channel (see Figure 1, main paper), this ‘natural backfill’ has a large component of fine sediments (including clay) due to the oxbow lake deposits above the paleo-channel (Figure 4, main paper). The borehole flowmeter tests from the paired wells located outside the paleo-channel boundaries produced similar hydraulic conductivity profiles. For the well pairs inside the paleo-channel boundaries, however, the ‘natural backfill’ and ‘gravel pack’ wells produced significantly different hydraulic

conductivity profiles. For instance, at well location 73 (see Figure A-1), most of the hydraulic conductivity values above 59 m msl for the gravel-packed well are 10 to 1000 times greater than those for the natural-backfilled well.

Young (1998) and Young et al. (1998) attribute the lower hydraulic conductivity values for the natural-backfilled well to two factors. One factor is that the clayey oxbow deposits (Figure 4, main paper) in the natural backfill partially block flow into the well from the highly permeable deposits at elevations from 59 m to 62 m msl. The other factor is that the Cooper-Jacob method used by Rehfeldt et. al (1989 and 1992) to calculate transmissivity values is based on a single measurement of (final) drawdown and an assumed storativity value whereas the Cooper-Jacob straight line method used by Young (1998) and Young et al. (1998) only considers a late time slope of a semilog plot of drawdown versus time. Another difference between the two methods is that whereas Rehfeldt et al. (1989 and 1992) measure drawdown from manually measured water levels, Young (1998) and Young et al. (1998) determine the late time slope from water levels measured using a transducer and a datalogger.

Table A-1 compares several metrics among the four well pairs located inside the paleo-channel. A key feature is the differences in transmissivity values across well installation types and by the analysis method. The gravel-packed wells have more than double the average specific capacity

and the average transmissivity values, as calculated using the Cooper-Jacob Equation, than do the natural-backfilled wells. For the Cooper-Jacob straight-line method, which calculates transmissivity independent of total drawdown, the average transmissivity for the two gravel-packed and the natural-backfilled wells is within about 1%.

Table A-1. Average performance metrics for the four sets of paired wells located inside the paleo-channel

Performance Metric	Filter Packed (A)	Natural Backfill (B)	Ratio (A/B)
Percent of total flow above 59 m elevation (channel deposits)	46%	9%	5.1
Specific capacity (Lpmin/m)[1]	75.2	34.5	2.2
Transmissivity calculated using the Cooper-Jacob Equation[1]	12.1	4.6	2.6
Transmissivity calculated using the Cooper-Jacob Straight-line method[1]	42.0	42.6	0.99
[1]Values are from Young (1998)			

In summary, Young (1998) and Young et al. (1998) identify significant sources of bias/error with the method used by Rehfeldt et al. (1989 and 1992) to determine borehole flowmeter hydraulic conductivity values. This bias is attributed to the well installation method, the testing method, and the analysis method used. The natural backfill method created a positive skin effect for wells in the paleo-channel, the testing methods did not detect the positive skin effect, and the analysis method using the Cooper-Jacob Equation does not account for the impact that positive skin effects

can have on calculated transmissivity values based on drawdown data from the production well.

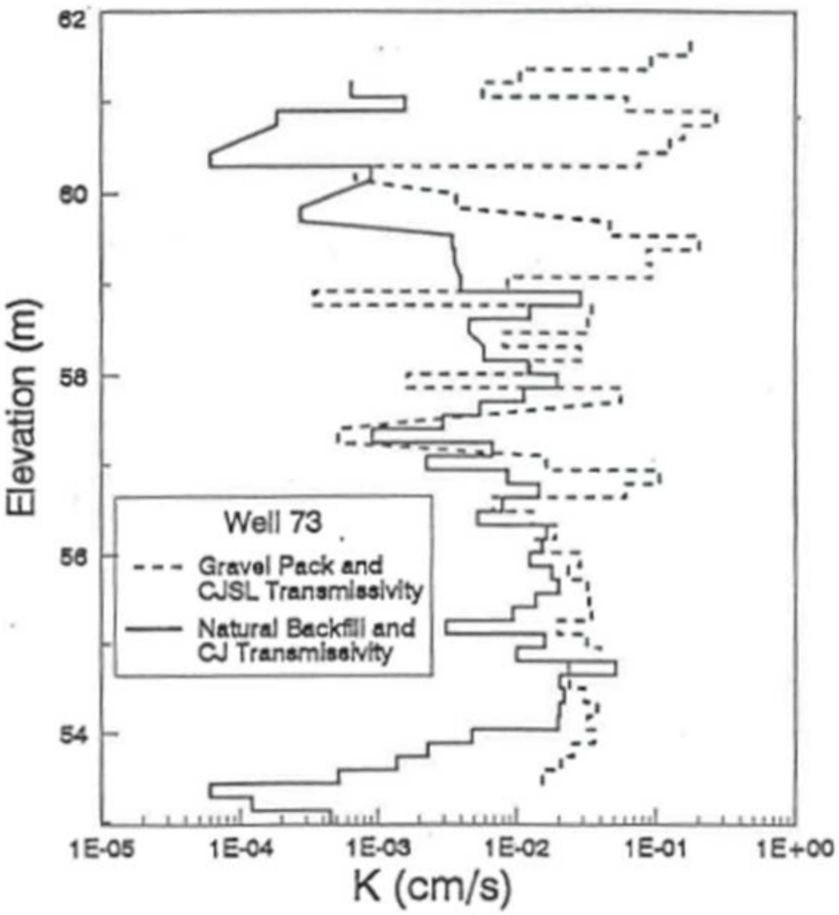

Figure A-1. Comparison of flowmeter K values from gravel-packed and natural-backfilled wells at location 73 inside the paleo-channel (from Young 1998). Note that CJSL Transmissivity results from Cooper-Jacob straight line method used by Young (1998) and Young et al. (1998) and CJ Transmissivity results from Cooper-Jacob Equation method Rehfeldt et. al (1989 and 1992)

Supplementary information B

Representativeness of the Tracer Concentrations from the Multi-level Samplers used at MADE

For some research projects, what is not published can be just as important, if not more important, than what is published. This situation is the case for the documentation of the Multi-Level-Sampler (MLS) testing at the MADE site (Boggs et al. 1988). A MLS is essentially a 0.025 cm PVC pipe filled with 0.016 m outer diameter (OD) tubes that exit through holes drilled in the pipe at different depths and with the open ends of the tubes wrapped in nylon filter cloth. At the MADE site, the majority of the MLSs were installed in a borehole drilled using a 0.18 m OD hollow-stem auger. Boggs et al. (1992) provide the following summary of the 3-year effort that resulted in selecting an augered borehole for MLS installation at the MADE site:

> “The field performance of the driver and augered MLS was evaluated prior to the natural gradient tracer experiment (Boggs et al. 1988). Small-scale tracer tests were conducted on both types of samples to investigate cross-talk between sampling ports during the sample collection and short-circuiting of groundwater in the well annulus under ambient conditions. Over 85% of the tests performed on the augered MLS show no evidence of cross-talk or short-circuiting. The driven MLS showed about the same level of performance for the cross-talk tests, but evidence of short-circuiting was indicated in approximately 40% of the tests. On this basis the augered MLS was selected as the primary design for the sampling

network."

A concern regarding the summary by Bogg et al. (1988) is that it omits a considerable amount of information assembled over the three-year investigation period that is relevant to the question of whether the MLSs are performing satisfactorily. The initial small-scale tracer testing of the MLSs at the MADE site was similar in design to two of the small-scale tracer tests performed between MLS ports in a single borehole prior to a natural-gradient test at Otis AFB, Massachusetts (LeBlanc et al. 1987). These two single-borehole MLS small-scale tracer tests at Otis AFB are illustrated in Figure B-1, upper right side. The two tests involved injecting a 400 milliLiter tracer pulse into a sampling port followed by injecting water continuously at 320 milliLiter /minute while withdrawing water from a single port vertically spaced 0.25 m away at a rate between 220 and 320 milliLiter /minute (Garabedian et al. 1988). At the Otis test site, the initial detection of the tracer occurred after withdrawing between 2.4 and 5.7 Liters from the single port. Maximum concentration of between 13% and 5% of the initial concentrations occurred after withdrawing 3.3 Liters and 8 Liters (i.e., after withdrawing approximately 10-20 minutes). The third tracer test (Figure B-1, lower left) involved injecting a tracer slug of 1000 mL and then withdrawing from only one adjacent port at a rate of 325 mL/min. The peak concentration occurred after 10 minutes, after withdrawing 3.25 Liters (Figure B-1, lower right, for a schematic of the third tracer test).

Supplementary information B

After visiting the Otis Test site and reviewing the MSL testing method as described above, the second author of this paper performed similar small-scale tracer tests between MLS ports in a single borehole at the MADE site, and the results were drastically different. At the ports adjacent to the injection port, concentrations much greater than 15% were typically realized in the first 50 ml volume sampled, and often the tracer was detected at more than one adjacent port.

After performing numerous MLS tracer tests in several boreholes over several months and using a range of tracer volumes and injection schemes, a final test procedure was established at the MADE site. This procedure was very different from the procedures used at Otis AFB. Two of these changes were: 1) the tracer volume was reduced from several hundreds of milliLiter s to just 3.2 milliLiter (approximately a teaspoon); and 2) during the withdrawal of groundwater after injecting the tracer slug, the port used for the tracer injection is used to withdraw tracer instead of continually injecting as per the above-described procedure at Otis AFB.

Figure B-1, left hand side, illustrates the mechanics of small-scale tracer tests performed at MADE and summarized by Boggs et al. (1988). These tracer tests were divided into two groups. Group 1 -

the cross-talk tests - involved withdrawing 50 ml volumes from the ports five minutes after injecting the tracer slug. Group 2- the short-circuiting tests were identical to the Group 1 tests except that the withdrawal of groundwater from the ports occurred one to twenty-two hours after injecting the tracer slug.

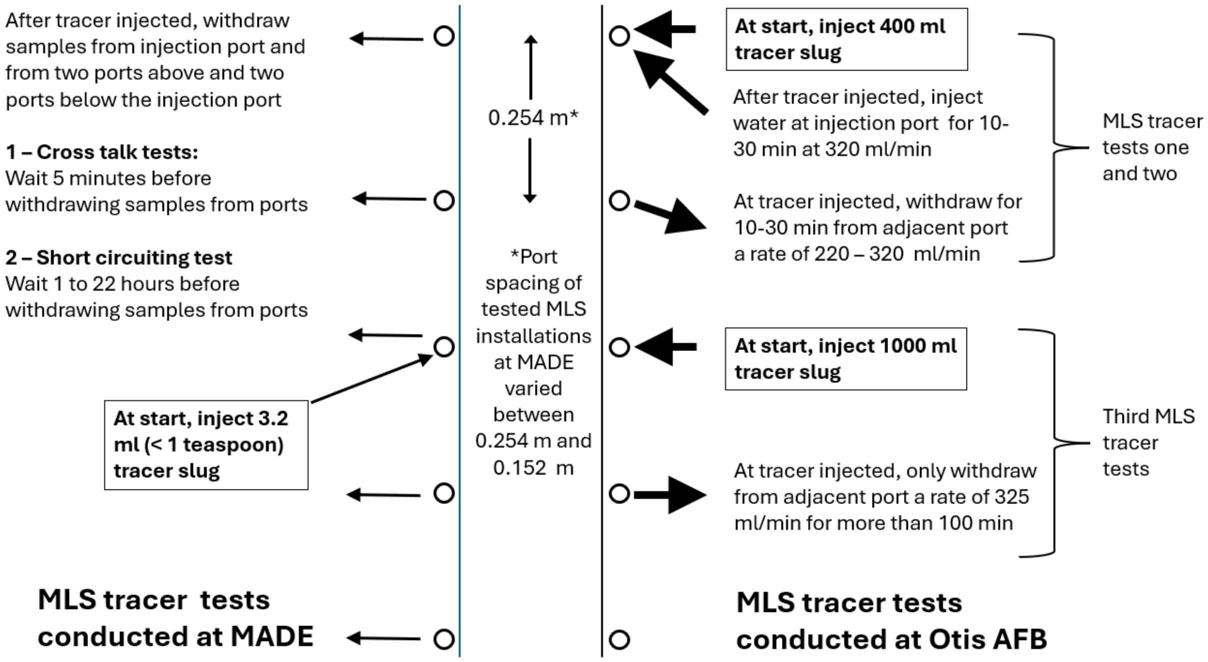

Figure B-1. Schematic of single well MLS port testing. Right – tests conducted at Otis AFB (Garabedian et al. 1988); Left – test conducted at MADE as reported by Boggs et al. (1988).

A significant shortcoming of Boggs et al. (1988) is that only results from the small-scale tracer tests with a tracer volume of 3.2 milliLiter were reported. Another significant shortcoming is that Boggs et al. (1988) made no attempt to model whether small-scale tracer tests could provide meaningful data on whether the samplers

were performing adequately. As mentioned previously, tracer tests with volumes greater than 3.2 milliLiter were conducted at the onset of MLS testing but were soon discontinued due to rapid spread between ports. Several articles published on the MADE tests cite Boggs et al. (1988) when discussing the reported mass balance error and sampling accuracy at MADE. The authors of this paper have noted that none of these articles, however, question whether or not the MADE cross-talk tests, which inject a tracer slug of about one-thousandth the size used to test MLS samples at Otis AFB, are a meaningful test for evaluating if the MLS would properly sample the tracer plume in three dimensions.

Two factors that likely contributed to the radically different small-scale tracer test results at the MADE site compared with those at the Otis test sites are the vertical hydraulic gradients and the composition of the aquifer deposits. The aquifer at Otis AFB test sites consists of clean, well-sorted, cohesionless sand that reportedly collapses readily around the MLS after installation. The aquifer at the MADE site consists of highly heterogeneous clays, sands, and gravel mixtures that do not readily collapse around the MLS after installation. Whereas Otis AFB test sites exhibit negligible vertical hydraulic gradients between sampling ports, vertical hydraulic gradients exceeding 5% are common between MLS ports at the MADE site. The main paper (Figure 7) shows the spatial trend

of these vertical gradients between the lower and upper parts of the aquifer. Figure B-2 shows additional detail of vertical gradients, based on heads measured between ports on a MLS located at MADE using a vacuum manifold manometer (Young, 1988) connected to an individual MLS. The measured hydraulic heads in each MLS are presented as head differences relative to the hydraulic head in the lowest sampling port. The vertical gradients were measured at 15 MLSs (Boggs et al. 1988) near the MADE wells used for the initial tracer injection. The detected tracer migration to adjacent ports in all small-scale tracer tests was consistent with the direction of the hydraulic gradient.

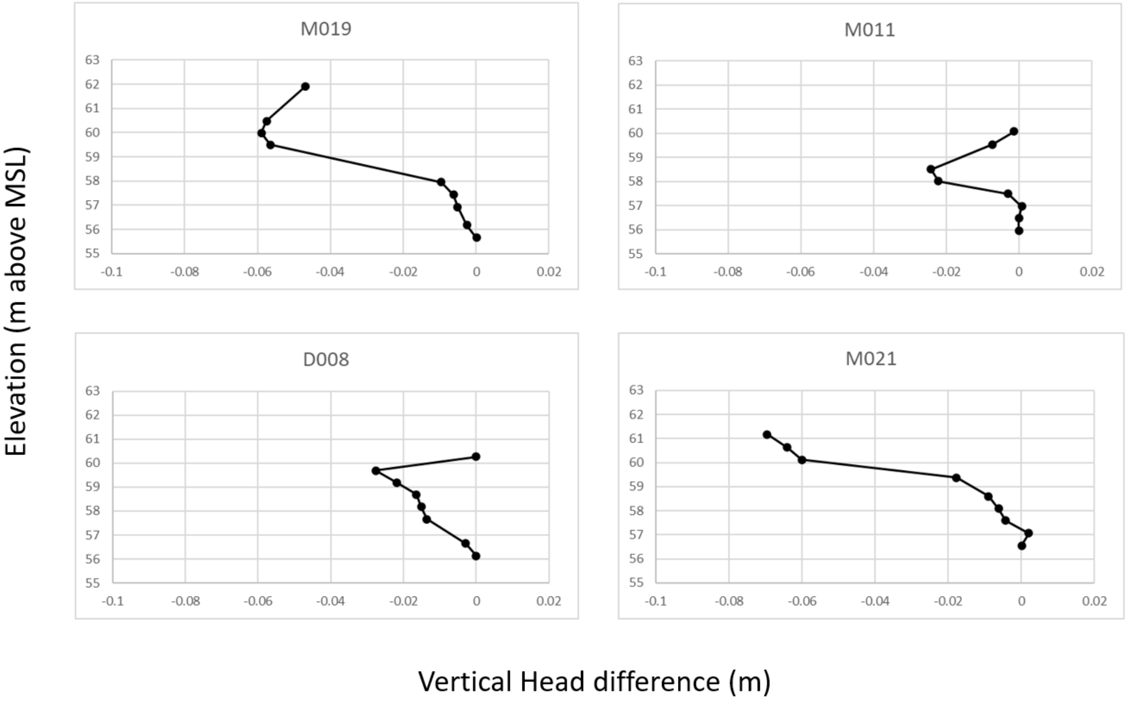

Figure B-2. Vertical hydraulic gradients (April 1987) for MLS wells, presented as head differences relative to the head in the lowest sampling port (from Boggs et al. 1988).

Supplementary information B

Based on the above, we believe that a substantial number of concentrations measured from samples collected by MADE MLSs are not representative of the concentrations in the undisturbed aquifer. These bias concentrations should be considered a potentially important contributor to the mass balance problems in the MADE tracer tests until analyses show otherwise. The complexity of the issues discussed in this supplement, in combination with the vertical gradients, makes it also quite unlikely that the concentration profiles at a MLS above can be mediated by averaging and normalization as proposed by Adams and Gelhar (1992) and used subsequently by many papers on MADE.

Supplementary information - References

Adams, E.E., and L.W. Gelhar. 1992. Field study of dispersion in a heterogeneous aquifer. 2. Spatial moments analysis. *Water Resources Research* 28, no. 12: 3293–3307. https://doi.org/10.1029/92WR01757.

Boggs, J.M., S.C. Young, H.F. Hemond, L. Richardson, and M.E. Schaefer. 1988. Evaluation of tracer sampling devices for the macrodispersion experiment. Research Report EA-5816, EPRI, Palo Alto CA USA.

Boggs, J.M., S.C. Young, L.M. Beard, L.W. Gelhar, K.R. Rehfeldt, and E.E. Adams. 1992. Field study of dispersion in a heterogeneous aquifer: 1. Overview and site description. *Water Resources Research* 28, no. 12: 3281–3291, https://doi.org/10.1029/92WR01756.

Bohling, G.C., G. Liu, P. Dietrich, and J.J. Butler. 2016. Reassessing the MADE direct-push hydraulic conductivity data using a revised calibration procedure. *Water Resources Research* 52, no. 11: 8970–8985. https://doi.org/10.1002/2016WR019008.

Garabedian, S.P., L.W. Gelhar, and M.A. Celia. 1988. Large-scale Dispersive Transport in Aquifers: Field Experiments and Reactive Transport Theory. MIT, Ralph M. Parsons Laboratory Hydrology and Water Resources Systems, Cambridge MA USA. https://dspace.mit.edu/handle/1721.1/143063.

LeBlanc, D.R., Garabedian, S.P., Wood, W.W., Hess, K.M. and Quadri, R.D., 1987, Natural-gradient tracer test in sand and gravel: Objective, approach, and overview of tracer movement in U.S. Geological Survey Open-File Report 87-109, B9-12.

Rehfeldt, K.R., P. Hufschmied, L.W. Gelhar, and M.E. Schaefer. 1989. Measuring hydraulic conductivity with the borehole flowmeter. Report EN-6511, EPRI, Palo Alto CA USA.

Rehfeldt, K.R., J.M. Boggs, and L.W. Gelhar. 1992. Field study of dispersion in a heterogeneous aquifer: 3. Geostatistical analysis of hydraulic conductivity. *Water Resources Research* 28, no. 12: 3309–3324. https://doi.org/10.1029/92WR01758.

Young, S. C., 1995. Application of Aquifer Tests and Sedimentology

Concepts to Characterize the Hydrological Properties of a Fluvial Deposit. Dissertation: University of Waterloo, Ontario, Canada. Pg 405.

Young, S. C., 1988. Development of a Driven Multilevel Groundwater Sampling Well and Instrumentation of Measuring Vertical Hydraulic Gradients over Short Distances. DOE Model Conference Proceedings, DOE/Oak Ridge Operations and Martin Maarietta Energy Systems, pg 93-104

Young, S.C. 1998. Impacts of positive skin effects on borehole flowmeter tests in a heterogeneous granular aquifer. *Groundwater* 36, no. 1: 67–75. https://doi.org/10.1111/j.1745-6584.1998.tb01066.x.

Young, S.C. H.E. Julian, H.S. Pearson., F.J. Molz, and J.K. Bowman. 1998. Application of the Electromagnetic Borehole Flowmeter, Report EPA/600/R-98/058, USEPA, Ada, OK.

Zech, A., S. Attinger, A. Bellin, V. Cvetkovic, G. Dagan, M. Dentz, P. Dietrich, A. Fiori, and G. Teutsch. 2021. A comparison of six transport models of the MADE-1 experiment implemented with different types of hydraulic data. *Water Resources Research* 57, no. 5: e2020WR028672. https://doi.org/10.1029/2020WR02867
